# Cerebrovascular Autoregulation in Preterm Infants During and After Surgical Ligation of the Ductus Arteriosus, a Comparison Between Two Surgical Approaches

**DOI:** 10.3389/fped.2020.00334

**Published:** 2020-07-10

**Authors:** Elisabeth M. W. Kooi, Michelle E. van der Laan, Ryan E. Accord, Marcus T. R. Roofthooft, Marcel J. Aries, Jan Willem J. Elting

**Affiliations:** ^1^University of Groningen, University Medical Center Groningen, Beatrix Children's Hospital, Division of Neonatology, Groningen, Netherlands; ^2^University Medical Center Groningen, University of Groningen, Center for Congenital Heart Diseases, Groningen, Netherlands; ^3^University Medical Center Groningen, University of Groningen, Center for Congenital Heart Diseases, Beatrix Children's Hospital, Division of Pediatric Cardiology, Groningen, Netherlands; ^4^University of Groningen, University Medical Center Groningen, Center for Congenital Heart Diseases, Division of Cardiothoracic Surgery, Groningen, Netherlands; ^5^University of Maastricht, Maastricht University Medical Center, Department of Intensive Care, Maastricht, Netherlands; ^6^University Medical Center Groningen, University of Groningen, Department of Neurology, Groningen, Netherlands

**Keywords:** cerebrovascular autoregulation, posterolateral thoracotomy, sternotomy, ductus arteriosus, ligation, cerebral hemodynamics

## Abstract

**Objective:** During ligation of the ductus arteriosus, cerebrovascular autoregulation (CAR) may deteriorate. It is unknown whether different surgical approaches affect changes in CAR differently. The objective of this study was to compare the potential change in CAR in preterm infants during and after ligation comparing two surgical approaches: sternotomy and posterolateral thoracotomy.

**Design:** This was an observational cohort pilot study.

**Setting:** Level III NICU.

**Patients:** Preterm infants (GA < 32 weeks) requiring ductal ligation were eligible for inclusion.

**Interventions:** Halfway the study period, our standard surgical approach changed from a posterolateral thoracotomy to sternotomy. We analyzed dynamic CAR, using an index of autoregulation (COx) correlating cerebral tissue oxygen saturation and invasive arterial blood pressure measurements, before, during, and after ligation, in relation to the two approaches.

**Measurements and Main Results:** Of nine infants, four were approached by thoracotomy and five by sternotomy. Median GA was 26 (range: 24.9–27.9) weeks, median birth weight (BW) was 800 (640–960) grams, and median post-natal age (PNA) was 18 (15–30) days, without differences between groups. COx worsened significantly more during and after thoracotomy from baseline (Δρ from baseline: during surgery: Δ + 0.32, at 4 h: Δ + 0.36, at 8 h: Δ + 0.32, at 12 h: Δ + 0.31) as compared with sternotomy patients (Δρ from baseline: during surgery: Δ + 0.20, at 4 h: Δ + 0.05, at 8 h: Δ + 0.15, at 12 h: Δ + 0.11) (*F* = 6.50; *p* = 0.038).

**Conclusions:** In preterm infants, CAR reduced significantly during and up to 12 h after ductal ligation in all infants, but more evident during and after posterolateral thoracotomy as compared with sternotomy. These results need to be confirmed in a larger population.

## Introduction

Cerebrovascular autoregulation (CAR) is often disturbed in preterm infants and can be assessed continuously using near-infrared spectroscopy (NIRS) (1).

During surgical ligation of a hemodynamically significant patent ductus arteriosus (hsPDA) in preterm infants, CAR may deteriorate unnoticed (2, 3), increasing the risk for silent hypoxic–ischemic cerebral injury due to hypoperfusion (4). Several studies that focused on long-term outcome have shown an association between surgical ligation of an hsPDA and neurodevelopmental impairment (5).

In March 2012, we changed our standard surgical approach from a posterolateral thoracotomy to a sternotomy. Both approaches have previously been compared in our preterm population, with lower post-operative pulmonary complications in the sternotomy group (6). Contrarily, in adult patients with congenital heart repair, the lateral approach showed a favorable intubation time and post-operative hospital stay (7). Effect on cerebral perfusion has not been investigated for both infants and adults.

During thoracotomy for ductal ligation in preterm infants, cardiac output has been shown to reduce, and systemic vascular resistance to increase, possibly due to pulmonary venous congestion while manipulating the lung (8, 9). Sternotomy on the other hand results in minimal to no manipulation of the lungs. Theoretically, this approach may therefore cause fewer fluctuations in cardiac output and therefore ensure a more stable cerebral perfusion.

While impaired CAR has been described in preterm infants undergoing surgery (10) and more specifically during thoracotomy, it is unknown whether CAR worsens after ductal ligation using a sternotomy. The aim of this study was to investigate the course of CAR during and after ductal ligation in preterm infants and to compare this course between the two surgical approaches.

## Materials and Methods

This was a retrospective observational cohort pilot study. All preterm infants born <32 weeks of gestational age (GA) requiring ductal ligation between July 2011 and September 2014 were considered eligible for inclusion. Only infants with routine cerebral NIRS (INVOS 5100C near-infrared spectrometer and neonatal SomaSensors, Covidien, Mansfield, MA, USA) and invasive arterial blood pressure (ABP) measurements for at least 1 h before, during, and at least 12 h after ligation were included. Both monitoring techniques are considered standard of care during ductal ligation in our center, but inserting a peripheral arterial line for continuous ABP measurement was not always possible. A post-ductal arterial line insertion was preferred.

Patients undergoing a posterolateral thoracotomy were placed in a right lateral decubitus position. The left chest was entered via a standard left posterolateral muscle splitting thoracotomy in the fourth intercostal space. The left lung was gently retracted, and the mediastinal pleura was incised over the proximal descending aorta. Then, the mediastinal pleural leaf was retracted to expose and identify the PDA, which was circumferentially dissected free and closed with at least one titanium hemoclip. The incised mediastinal pleura was closed, and the chest was routinely drained with a small pleural catheter and closed in layers after local anesthetic infiltration. For the median approach, a full median sternotomy was used for entering the chest and both pleurae were kept intact as much as possible. Next, the cranial portion of the pericardium was opened and gently suspended with traction sutures. The PDA was dissected free and closed with at least one titanium hemoclip. A single chest tube was placed through a stab-wound incision to drain the anterior mediastinum. The sternum was closed in standard fashion. For anesthesia, all infants regardless of the surgical approach received a standard combination of midazolam, fentanyl, and rocuronium. All operations were performed in our level III neonatal intensive care unit (NICU).

Regional cerebral tissue oxygen saturation (rcSO_2_) measured by NIRS and ABP data were collected with a sample frequency of 0.2 Hz and stored offline for analysis. Data artifacts were removed with linear interpolation and by applying a median filter. Dynamic CAR was quantified using a previously described index of autoregulation (COx, or TOx), which is a correlation coefficient (ρ) between 10-s averaged values of rcSO_2_, and mean ABP (MABP) over moving 5-min time windows (using MATLAB R2007a, MathWorks, Natick, MA, USA) (1, 11, 12). A moving-average value was created with maximal overlap, i.e., a new COx value was calculated every 10 s. An increase in the COx correlation coefficient is interpreted as reduction in CAR capacity.

We averaged the COx values over the following time periods: (1) pre-ligation (max 4 h, referred to as “baseline”), (2) during ligation, (3) 0–4 h after ligation, (4) 4–8 h after ligation, and (5) 8–12 h after ligation. We used SPSS 22.0 (IBM Corp., Armonk, NY, USA) for descriptive statistics and the Friedman test to assess changes in clinical parameters. Changes in COx over time and between surgical approaches were evaluated using repeated measurements ANOVA. The protocol was approved by the local UMCG ethics committee. According to Dutch medical law, written informed consent was deemed unnecessary by the ethics committee, as routinely collected clinical data were anonymously analyzed.

## Results

Between July 2011 and September 2014, 27 ductal ligations were performed in infants with a GA <32 weeks. Nine infants had complete datasets; the first four were approached via lateral thoracotomy, the latter five via sternotomy. Median GA was 26 (range: 24.9–27.9) weeks, median birth weight (BW) was 800 (640–960) grams, and median post-natal age (PNA) was 18 (15–30) days. There were no differences between both groups regarding GA, BW, PNA, baseline MABP, FiO_2_, and mean airway pressure (MAP) (Table 1).

**Table 1:** Baseline characteristics of the infants that underwent ductal ligation via posterolateral thoracotomy or via sternotomy.

**Characteristic**	**Posterolateral thoracotomy**	**Sternotomy**	***p*-value**
GA (weeks)	26.7 (25.6–27.9)	26.0 (24.9–26.0)	0.80
BW (gram)	820 (640–960)	800 (670–960)	1.0
PNA (days)	17.5 (14–30)	18 (15–24)	0.62
IVH > grade 1	0	0	–
Baseline MABP (mmHg)	32 (30–38)	32 (28–33)	0.33
Baseline rcSO_2_ (%)	68 (62–73)	57 (52–65)	0.05
Baseline COx (ρ)	−0.38 (−0.52 to −0.10)	−0.01 (−0.14–0.07)	0.003
Duration of surgery (min)	55 (33–80)	84 (70–150)	0.05
Hemoglobin (<12 h before surgery) (mmol/l)	7.9 (7.7–8.1)	8.4 (7.4–8.9)	0.41
FiO_2_ baseline (%)	37 (28–45)	34 (28–38)	0.56
MAP baseline (cm H_2_O)	6.8 (6.6–7.7)	6.4 (6.1–7.7)	0.41

*MAP, mean airway pressure; FiO_2_, fractional inspired oxygen; IVH, intraventricular hemorrhage. Data are presented as median (range)*.

The thoracotomy group tended to have lower baseline rcSO_2_ and shorter duration of surgery. We did not find a statistically significant change in mean MABP (*p* = 0.24) or rcSO_2_ (*p* = 0.09) when comparing the various study periods within the total group.

**Figure 1:**
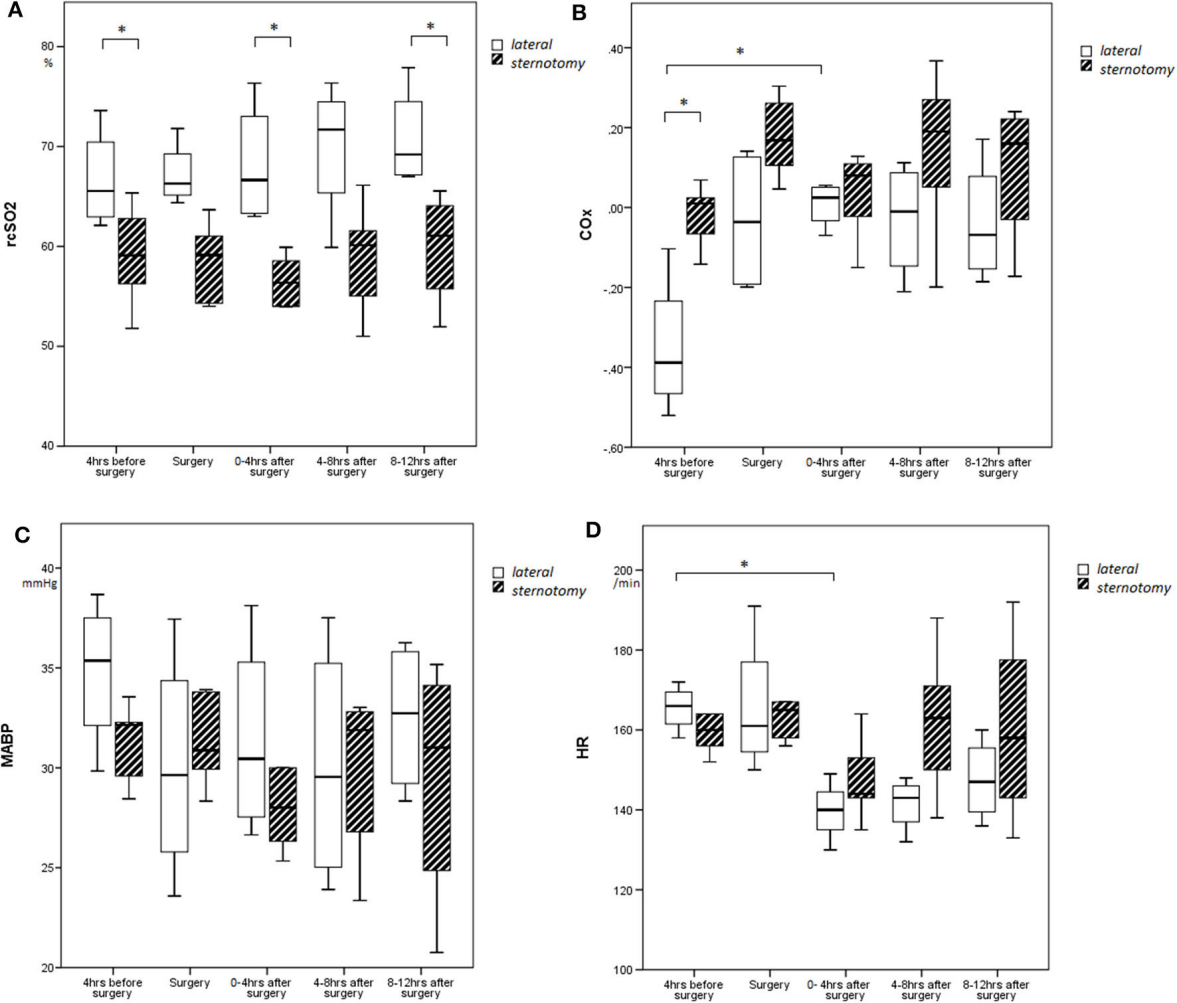
Course of **(A)** rcSO_2_, **(B)** COx, **(C)** MABP, and **(D)** heart rate over time depicted by boxes and whiskers, representing medians, interquartile ranges, and min/max. *p < 0.05.

After surgery, we found no difference in MABP values and FiO_2_ levels between both groups, and a similar difference in rcSO_2_ as observed before surgery between both groups. Both groups demonstrated a drop in heart rate after surgery, but not statistically different between both groups (*p* = 0.62). More vasoactive drugs were administered after surgery in the sternotomy group for low blood pressure (Table 2, Figure 1).

**Table 2:** Values during and after surgery of the infants that underwent ductal ligation via posterolateral thoracotomy or via sternotomy.

**Parameter**	**Posterolateral thoracotomy**	**Sternotomy**	***p*-value**
MABP (mmHg) after surgery	30.3 (26.0–36.6)	28.0 (25.2–39.1)	0.62
rcSO_2_ (%) after surgery	67.0 (62.9–75.1)	56.3 (48.3–60.3)	0.014
CO_2_ (kPa) <1 h after surgery	6.6 (4.7–7.5)	5.1 (3.1–7.7)	0.56
FiO_2_ after surgery (%)	45 (21–51)	37 (25–40)	0.41
Vasoactive medication during/after medication	1/4 (25%)	4/5 (80%)	0.10

*MAP, mean airway pressure; FiO_2_, fractional inspired oxygen; NR, not recorded. Data are presented as median (range)*.

**Figure 2:**
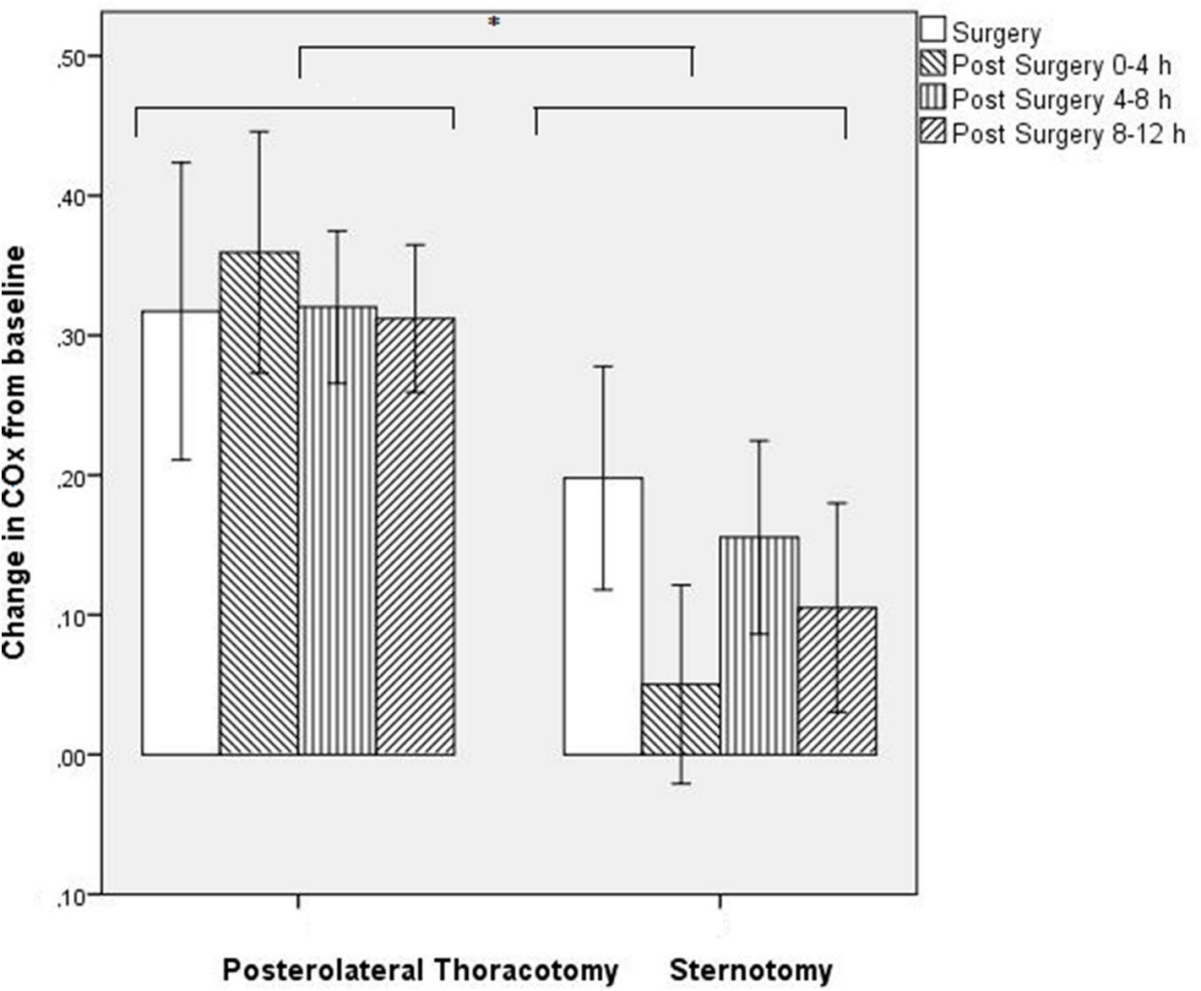
Change in COx value (ρ) from baseline for both groups separately. Mean ± S.E.M. *p < 0.05.

COx values changed significantly over time (*F* = 9.95; *p* = 0.024), with higher COx values during and after surgery as compared with baseline for all defined time periods (Figure 1). Although the two surgical groups differed in baseline COx, the thoracotomy group showed a significantly higher increase in COx from baseline (Δρ from baseline: during surgery: Δ + 0.32, 4 h: Δ + 0.36, 8 h: Δ + 0.32, 12 h: Δ + 0.31) as compared with the sternotomy group (Δρ from baseline: during surgery: Δ + 0.20, 4 h: Δ + 0.05, 8 h: Δ + 0.15, 12 h: Δ + 0.11) (*F* = 6.50; *p* = 0.038) (Figure 2). The results remained the same after correcting for gestational age.

## Discussion

CAR capacity assessed using the non-invasive NIRS based COx index, reduced during and up to 12 h after ductal ligation compared to baseline. Patients operated via a posterolateral thoracotomy showed significantly more worsening of autoregulation (>30% reduction of CAR), as compared with patients that underwent sternotomy (<20% reduction). Even in this small sample and after correction for multiple comparisons, these effects remained significant.

Our findings confirm previously described worsening of CAR during and after ductal ligation via thoracotomy (2, 3), which may be due to anesthetics on the one hand, which applies for both approaches, and to changes in intrathoracic pressures on the other hand, which applies especially for the thoracotomy approach. Whether the absolute COx values found in our population confirm impaired CAR is still under debate. Positive COx levels suggest blood pressure dependent cerebral oxygenation, with higher levels relating to clinical adverse outcome (1, 11).

A few studies have demonstrated impaired CAR during and after thoracotomy for the correction of congenital heart defects. These studies mostly concern older infants and children with more matured cerebral vasculature, in whom we assume sternotomy was most commonly used (not described). In these infants and children, impaired CAR during and after thoracic surgery has been attributed to hypotensive episodes, induced hypothermia, and increased end-tidal pCO_2_ values (12, 13). Although the sternotomy approach took longer in our population, we expect no large differences in temperature between the two groups in our study but could not confirm this, retrospectively. All infants remained in their incubator with the top off, and an external heater added. Also, MABP and pCO_2_ was not different between groups.

We observed that during thoracotomy, CAR worsens more than during sternotomy. We offer several explanations for this finding. Using thoracotomy, the left lung is manually compressed to the side. We speculate that compressing the left lung may lead to a subsequent reduction in pulmonary venous return, resulting in a reduced cardiac output. Furthermore, infants receiving thoracotomy are turned over to their right side, whereas infants undergoing sternotomy remain in a supine position. The subsequent compression of the right lung will enhance this negative effect on pulmonary venous return during thoracotomy. During this phase of impaired cardiac output, cerebral perfusion pressure decreases and challenges adequate CAR in these immature and sedated infants. These intrathoracic pressure changes are probably less pronounced during sternotomy. This speculation, however, is not supported by our clinical data regarding FiO_2_ and CO_2_ levels, which were similar between the two approaches after surgery in the study group. Furthermore, more vasoactive medication was administered after sternotomy, resulting in MABP not being different between the groups, contradicting this speculation. We did notice a low rcSO_2_ and non-significant lower MABP in the sternotomy group, already before surgery, coinciding with higher baseline COx values, suggesting preexisting poorer CAR in these infants.

In order to prevent any form of open thoracic surgery for ductal ligation, as it is known to cause hemodynamic shifts and complications, percutaneous trans-catheter patent ductus arteriosus closure seems a promising alternative, though also not without complications in the smallest infants. A reduction in pulmonary complications and less use of post-operative inotropes have been described, but surgical and percutaneous closures have never been prospectively compared (14).

A limitation of our study is its retrospective nature, comparing two small historical cohorts. Apart from the surgical approach, we are not aware of other changes in clinical practice over the three study years, though subtle changes in care may have been overlooked. We did not find clinical factors that may explain these differences in change in COx values between the two surgical approaches; baseline FiO_2_ was similar between groups, as were other potential factors that may influence COx values, such as MABP, MAP, CO_2_, and Hb levels. Since all infants received similar sedation using local protocol, this can also not explain the difference that was found. The same holds true for the fact that the ductus closed, inducing sudden hemodynamic changes (15).

Baseline rcSO_2_ values were lower, and duration of surgery tended to be longer in the sternotomy group as compared with the thoracotomy group. We have no clear explanation why the infants receiving sternotomy already had lower baseline rcSO_2_, since other cardiorespiratory baseline variables were similar between both groups. Finally, we noticed the difference in baseline COx. In such a small sample, this may be caused by chance, for we cannot explain how this clinically would have influenced our results, though statistically, the infants undergoing thoracotomy had more room for deterioration.

Regarding the comparison of other short-term outcomes of the two approaches, we recently published a comparable post-operative mortality and more days on opioids but significantly less pulmonary complications in the sternotomy group, compared to the thoracotomy group (6). Further research with larger samples with long-term clinical follow-up will be necessary to determine whether a median sternotomy approach is associated with favorable long-term results.

## Conclusions

This is the first pilot study to assess the influence of the surgical approach on CAR in preterm infants undergoing ductal ligation. We hypothesize that during posterolateral thoracotomy, pulmonary venous congestion may cause secondary decreased cardiac output and cerebral perfusion pressure, which challenges cerebrovascular autoregulation. This effect was less evident during sternotomy. Although with limited numbers, and different baseline rcSO_2_ and COx indices, we hypothesize that the surgical approach in itself can influence cerebral autoregulation. A larger study is needed to confirm this hypothesis.

## Data Availability Statement

The datasets for this study will be made available and can be requested at any time via the corresponding author.

## Ethics Statement

The studies involving human participants were reviewed and approved by METc UCMG. Written informed consent from the participants' legal guardian/next of kin was not required to participate in this retrospective study in accordance with the national legislation and the institutional requirements.

## Author's Note

This manuscript is an expansion of its abstract presented at the 5th International Meeting on Cerebral Haemodynamic Regulation.

## Author Contributions

EK designed the study, collected the data, performed the statistical analyses, composed the first draft of the manuscript, and approved the final version. ML collected the data, critically evaluated the manuscript, and approved the final version. RA and MR helped interpret the data, critically evaluated the manuscript, and approved the final version. MA helped in designing the study and the data analyses, critically evaluated the manuscript, and approved the final version. JE performed the statistical analyses, critically evaluated the manuscript, and approved the final version.

## Conflict of Interest

The authors declare that the research was conducted in the absence of any commercial or financial relationships that could be construed as a potential conflict of interest.
